# Central and Peripheral Oxygen Distribution in Two Different Modes of Interval Training

**DOI:** 10.3390/metabo11110790

**Published:** 2021-11-18

**Authors:** Korbinian Sebastian Hermann Ksoll, Alexander Mühlberger, Fabian Stöcker

**Affiliations:** 1 Institute of Sport Sciences, Department of Human Sciences, Universität der Bundeswehr Munich, 85579 Neubiberg, Germany; 2 Professorship of Biomechanics in Sports, Department of Sport and Health Sciences, Technical University of Munich, 80992 Munich, Germany; 3 Prevention Center, Department of Sport and Health Sciences, Technical University of Munich, 80992 Munich, Germany; alex.muehli@freenet.de (A.M.); fabian.stoecker@tum.de (F.S.)

**Keywords:** interval exercise, oxygen uptake (VO_2_), cardiac output (CO), oxygen availability (HHb/VO_2_), near-infrared spectroscopy (NIRS)

## Abstract

In high-intensity interval training the interval duration can be adjusted to optimize training results in oxygen uptake, cardiac output, and local oxygen supply. This study aimed to compare these variables in two interval trainings (long intervals HIIT3m: 3 min work, 3 min active rest vs. short intervals HIIT30s: 30 s work, 30 s active rest) at the same overall work rate and training duration. 24 participants accomplished both protocols, (work: 80% power output at VO_2_peak, relief: 85% power output at gas exchange threshold) in randomized order. Spirometry, impedance cardiography, and near-infrared spectroscopy were used to analyze the physiological stress of the cardiopulmonary system and muscle tissue. Although times above gas exchange threshold were shorter in HIIT3m (HIIT3m 1669.9 ± 310.9 s vs. HIIT30s 1769.5 ± 189.0 s, *p* = 0.034), both protocols evoked similar average fractional utilization of VO_2_peak (HIIT3m 65.23 ± 4.68% VO_2_peak vs. HIIT30s 64.39 ± 6.78% VO_2_peak, *p* = 0.261). However, HIIT3m resulted in higher cardiovascular responses during the loaded phases (VO_2_ *p* < 0.001, cardiac output *p* < 0.001). Local hemodynamics were not different between both protocols. Average physiological responses were not different in both protocols owning to incomplete rests in HIIT30s and large response amplitudes in HIIT3m. Despite lower acute cardiovascular stress in HIIT30s, short submaximal intervals may also trigger microvascular and metabolic adaptions similar to HIIT3m. Therefore, the adaption of interval duration is an important tool to adjust the goals of interval training to the needs of the athlete or patient.

## 1. Introduction

Interval training is an often-used training modality to improve endurance performance in athletes but also cardiorespiratory fitness in patients [1,2,3]. In contrast to continuous training, interval training consists of several alternating phases of high and low intensities. Buchheit & Laursen [1] defined multiple exercise variables used in the design of an interval exercise session including intensities and durations of work and relief phases, the work modality, and the combination of exercise series. The manipulation of these factors adjusts the interval training in order to meet the demands of the sport, the athlete’s profile, or the patient’s possibilities [1]. Sprint interval training or repeated sprint interval training, at maximum effort, highly affect the capability in maximal energy production by aerobic and anaerobic systems while short (<45 s) and long (2–4 min) high-intensity interval training (HIIT) is associated with higher emphasis on submaximal performance [1,4]. Due to intermittent exercise of work and relief, HIIT achieves longer times at high rates of oxygen uptake (VO_2_) compared to long slow distance or moderate continuous training with the same training duration [1,5]. Accordingly, HIIT represents a greater stimulus on maximum aerobic energy production and hence is associated with a fast increase in peak oxygen uptake (VO_2_peak) [5,6]. Both, HIIT and continuous training affect the cardiovascular system by increasing local perfusion [7]. Microvascular oxygen distribution and capillary perfusion are known as key determinants to promote oxidative metabolism [8]. Recent studies showed evidence for a higher impact of interval training on local muscle perfusion compared to continuous training [9,10]. However, there are several confounding variables in the investigation of interval training applications. One major issue in the research of intermittent training is the matching of interval intensity and duration. Using an isoeffort matching approach, Zafeiridis and colleagues compared a continuous training (70% VO_2_max), HIIT with long intervals (2 min at 95% VO_2_max, 2min passive rest), and HIIT with short intervals (30 s at 110% VO_2_max, 30 s passive rest). Cardiovascular stress was highest in continuous and long interval training, while muscle oxygenation was equal in all protocols [11]. However, this study does not clarify the effect of different interval durations, as the work rate was not constant. Our study aimed to compare two interval regimens of equal overall work rate but the different composition of work- and relief-interval duration in respect of local and central cardiovascular effects. We hypothesized that long interval duration, i.e., 3 min work, has higher cardiometabolic demand compared to short interval duration, i.e., 30 s, at same overall work. Despite the different cardiometabolic demands, both interval protocols achieve similar effects in acute microvascular oxygen distribution.

## 2. Methods

24 male subjects (Table 1) participated in this study voluntarily. For this, informed consent was obtained from all subjects involved in the study. All test persons were healthy and performed recreational sport at least two times a week. This study was carried out in accordance with the Declaration of Helsinki and was approved by the local Ethics Committee of the Technical University of Munich (#67/14, 2014).

On an electrically braked cycle ergometer (Lode Excalibur, Groningen, NL, USA) the participants performed three tests protocols which had to be separated at least 48 h from each other and executed within two weeks. During each measurement, an open spirometry device, Cortex Metalyzer 3b (Leipzig, GER), analyzed breath-by-breath the respiratory gas exchange. For this, the participants were not allowed to consume food and caffeinated drinks three hours before the measurements. The first test, a continuous ramp test to exhaustion (3 min baseline measurement, 3 min warm-up at 50 W, 20 W/min increase), was accomplished to determine the VO_2_peak, the respiratory thresholds (gas exchange threshold, GET + respiratory compensation point, RCP), peak heart rate (HR_peak_), cardiac output (CO_peak_), stroke volume (SV_peak_) and maximum power output (PO_peak_). VO_2_peak, HR_peak_, CO_peak_, SV_peak and_ was defined as the highest value in the 30 s moving average of each parameter. Both, GET and RCP, were estimated using the modified V-slope method [12] by determining visual investigation of breakpoints in the plotted breath-by-breath data of carbon dioxide output (VCO_2_) vs. oxygen uptake (VO_2_) [12,13]. To increase the accuracy of GET and RCP, visual breakpoints in breath volume (VE) vs. time as well as in the equivalents of VE/VCO_2_ and VE/VO_2_ vs. time were also used for threshold detection. Additionally, VE vs. VCO_2_ provided further information about the RCP. Without wearing special bike shoes, the participants were allowed to choose a comfortable cadence above 60 rpm which had to be maintained. The incremental ramp test was terminated by a drop of the pedal frequency below 60 rpm.

**Figure 1:**
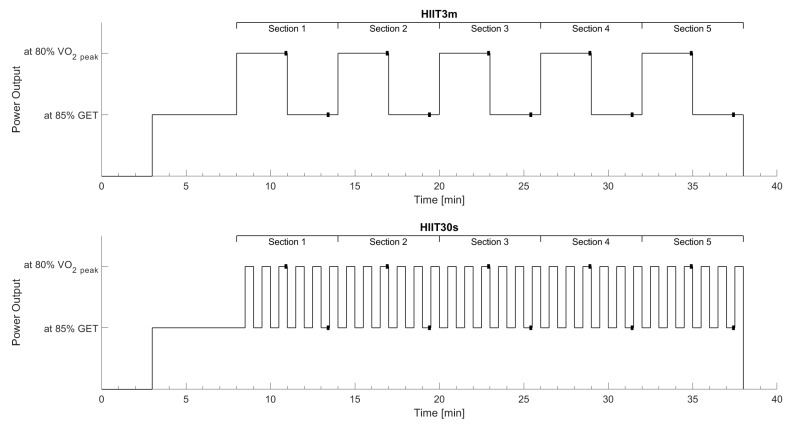
Scheme of HIIT3m and HIIT30s protocol regarding interval intensities and duration. The black squares demonstrate the 10 s period, where the means for work (loaded) and resting (unloaded) phases were calculated.

In randomized order, the participants performed two interval protocols that differed in the duration of the intervals. Each protocol consisted of five consecutive sections. In the long interval protocol (HIIT3m), one section was representing one complete interval including 3 min work phase and 3 min active rest. During the short interval protocol (HIIT30s), one section consisted of six repeated bouts of 30 s active recovery and 30 s work. The work-interval was set equal to the power output achieved at 80% of VO_2_peak in the incremental ramp protocol for both, HIIT3m and HIIT30s work intervals. The intensity was then reduced by 5% to account for the delayed oxygen kinetics i.e., a mean response time of about 30 s [14]. The recovery intensity was set to the power output achieved at 85% GET. Due to the work to rest ratio of 1:1 and the same power output during work and relief phases, both interval protocols achieved the same amount of total work (Figure 1). Each interval session began with a 3 min baseline measurement of oxygen uptake, cardiac output, and muscle deoxygenation followed by a 5 min warm-up. The warm-up intensity was set equal to the active recovery intensity. The short intervals started with active rest to ensure that the last 30 s of a HIIT3m work interval was time-aligned with a work interval in the HIIT30s. After the last work phase of both protocols, 20 µL of blood was taken from the right earlobe for end-exercise lactate diagnosis. The blood-filled capillaries were stored in reaction cups and mixed with 1000 µL of hemolyzing solution. The calibrated Biosen S-Line system (EKF-diagnostic GmbH, Barleben, GER) analyzed the probes using the enzymatic-amperometric principle.

In addition to the heart rate (HR), stroke volume (SV) and cardiac output (CO) were analyzed non-invasively via impedance cardiography (PhysioFlow Enduro, Manatec Biomedical, Folschviller, France). Also known as transthoracic-electrical-bioimpedance, impedance cardiography detects changes of thoracic impedance caused by the alternating heart volume by sending a low amperage high-frequency current through the thorax [15,16]. The method of impedance cardiography, respectively the PhysioFlow System was validated against the direct Fick method and/or thermodilution in patients [15,16,17] and exercise tests [18]. For this method, six electrodes must be placed at the subject’s neck and upper body. Previously, the skin was shaved and cleaned with skin preparation gel and alcohol to increase skin conductance and signal quality. After connecting the PhysioFlow, the blood pressure was measured and the device was calibrated. For calibration, the participants were asked to maintain a relaxed position on the ergometer. As soon as the signal stabilized, the software analyzed 30 heartbeats to calibrate the system. The PhysioFlow system was not available to the first seven participants during piloting. Hence, SV and CO were analyzed in only 17 subjects, whereas VO_2_, HR, and muscle oxygenation were measured in all 24 test persons.

Muscle oxygen saturation was analyzed by near-infrared spectroscopy (NIRS). In general, a NIRS device consists of two optodes. The first emits near-infrared light into human tissue. On its way through the tissue, light, i.e., photons, can be absorbed or scattered by specific chromophores like oxygenated (O_2_Hb) or deoxygenated hemoglobin (HHb). The second optode receives the remaining, attenuated light. According to the modified Lambert-Beer law, attenuation of light and therefore the concentration of the absorbing chromophores can be calculated by the logarithmic ratio between received and emitted light. Due to the unknown pathlength of the photons inside the tissue, a differential pathlength factor has to be included. In the used system, this factor was equal to 4 [19].

The near-infrared spectroscopy device (Portamon, Artinis medical Systems, Elst, NL) was placed at the shaved, right m. vastus lateralis similar to the SENIAM recommendations for EMG application [20]. The device emitted light (760 and 850 nm) continuously to the 35 mm distanced receiving optode, resulting in a penetration depth of approximately 17.5 mm [21]. To ensure that light penetrates muscle tissue, the skinfold at the NIRS position was measured by a caliper (Skinfold Caliper, Harpenden, Burgess Hill, UK). Limited by the continuous wave design of the device, only relative concentrations of O_2_Hb [µmol*L^−1^] and HHb [µmol*L^−1^] can be measured. The NIRS software (Oxysoft, Artinis medical Systems, Elst, NL, USA) started recording with 10 Hz measuring rate and additionally calculated the total amount of hemoglobin (tHb = O_2_Hb + HHb [µmol*L^−1^]) as well as the tissue saturation index (TSI = 100*O_2_Hb*tHb^−1^ [%]). Measured HHb was normalized from 0% (baseline) to 100% (maximum HHb response during ramp test) to be comparable between both interval protocols.

Microvascular oxygen distribution was estimated by ΔHHb per unit ΔVO_2_ (ΔHHb/ΔVO_2_). The ratio of normalized HHb to normalized VO_2_ (100% = VO_2_peak) reflects the continuous matching of O_2_ distribution and its utilization. In general, a better microvascular O_2_ distribution is indicated by a lower ΔHHb/ΔVO_2_ ratio. This method was used previously in relation to microvascular O_2_ adjustment during exercise on-transient [8,22] as well as a description of microvascular O_2_ availability during and after exercise [11,23,24].

All data were collected and processed by customized Matlab scripts (Mathworks, Natick, MA, USA). The program interpolated the breath-by-breath data measured by the spirometry and PhysioFlow to second-by-second values. NIRS data were downsampled to 1 Hz. Data were smoothed using a moving average with a 30 s-time window. All data were normalized to the maximum value in the preceding maximal ramp-test. The section averages (1 long complete interval, 6 short complete intervals) as well as the end values of the work phase (loaded) and the resting phase (unloaded) were statistically analyzed. These end values were defined as the mean of the last 10 s of the loaded or unloaded phase (Figure 1) respectively. Repeated measures ANOVAs with two factors (interval training, section) were executed in SPSS 23 (IBM, Armonk, NY, USA). Results were corrected by the Greenhouse–Geisser method if the assumption of sphericity, tested by Mauchly’s test of sphericity, was violated. For the prevention of α-error accumulation, the Bonferroni–Holm method was used. Furthermore, time above GET, time above 80% VO_2_peak, and average oxygen uptake were calculated to estimate the overall respiratory impact of the interval protocols. A paired *t*-test analyzed these parameters as well as end-exercise lactate for significant effects. The level of significance was set to *p* = 0.05.

## 3. Results

### 3.1. Aerobic Rate

**Figure 2:**
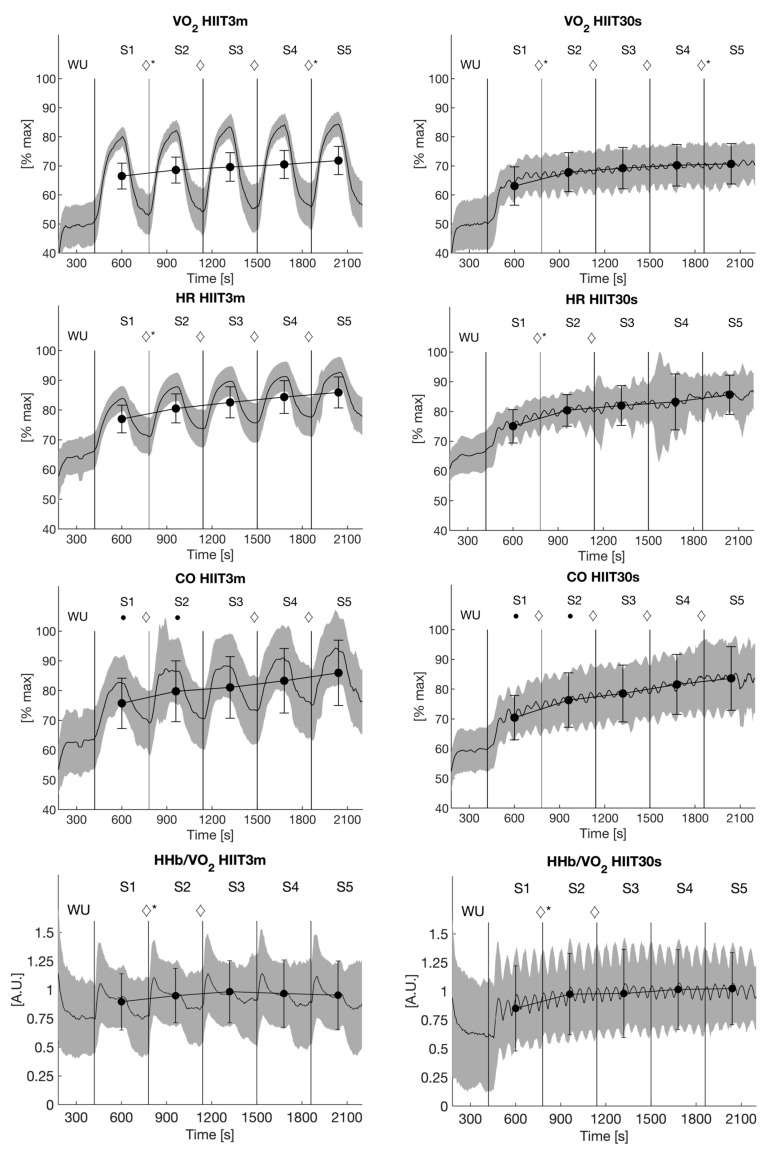
Averaged traces of relative oxygen uptake, heart rate, cardiac output and ΔHHb/VO_2_ ± SD (gray area); averaged values of each 6 min-section are presented as mean ± SD; ◊ indicate significant differences between two successive sections; interaction effects are marked with * (*p* ≤ 0.05).

Average oxygen uptake (Figure 2) increased progressively from Sections 1–5 in both protocols (HIIT3m: ANOVA *p* ≤ 0.001, post hoc tests *p* ≤ 0.001; HIIT30s: ANOVA ≤ 0.001, post hoc tests *p* ≤ 0.05). Although there was no significant difference in average oxygen uptake in any of the five sections between both protocols, there was a significant interaction effect between the first and second as well as the fourth and fifth section (ANOVA *p* ≤ 0.001, post hoc tests *p* ≤ 0.002).

**Figure 3:**
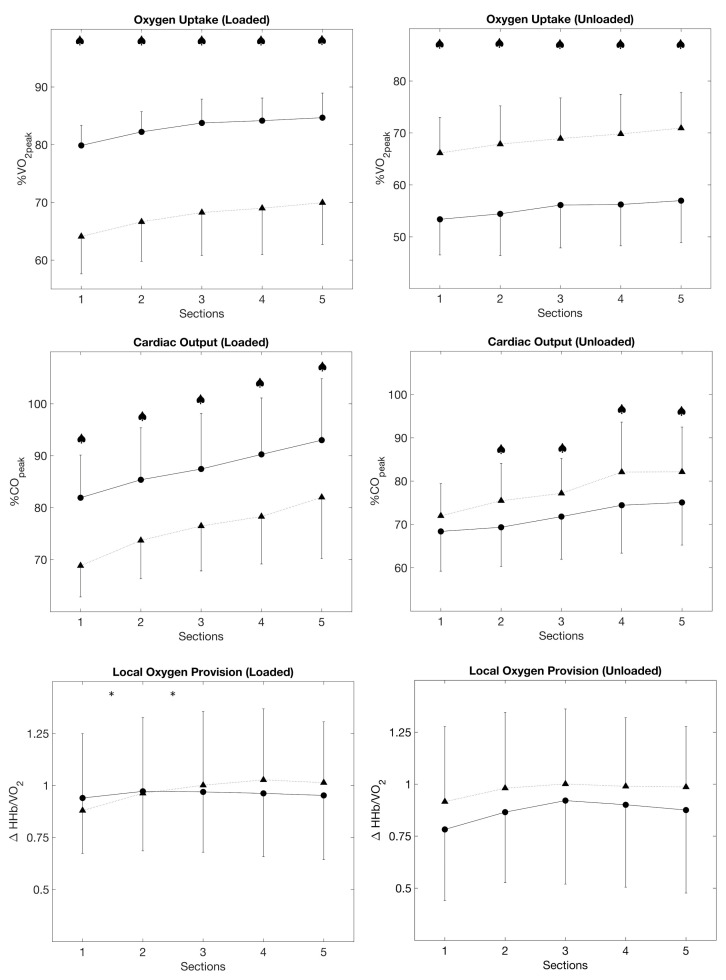
Averaged values for oxygen uptake, cardiac output and HHbVO_2_ at the end of loaded and unloaded phases for HIIT3m (•, solid line) and HIIT30s (▲, dashed line); ♠ indicates significant differences between both protocols (*p* ≤ 0.05); significant interaction effects are marked with * (*p* ≤ 0.05).

The loaded phases of all sections were significantly higher in the HIIT3m compared to HIIT30s (ANOVA *p* ≤ 0.001, post hoc tests *p* ≤ 0.001). During the unloaded phases, VO_2_ was higher in HIIT30s (ANOVA *p* ≤ 0.001, post hoc tests *p* ≤ 0.001, Figure 3).

### 3.2. Heart Rate

The average heart rate (Figure 2) also increased progressively from Sections 1–5 in HIIT3m (ANOVA *p* ≤ 0.001, post hoc tests *p* ≤ 0.001). In HIIT30s, relative HR increased up to Section 3 and flattened afterward (ANOVA *p* ≤ 0.001, post hoc tests *p* ≤ 0.05).

The loaded phases of all sections were significantly higher in HIIT3m compared to HIIT30s (ANOVA *p* ≤ 0.001, post hoc tests *p* ≤ 0.001). In contrast, HIIT30s resulted in higher HR during the unloaded phases (ANOVA *p* ≤ 0.001, post hoc tests *p* ≤ 0.001) (Figure 3).

### 3.3. Cardiac Output

Cardiac output (Figure 2) increased progressively with increasing exercise duration in both protocols (ANOVA *p* ≤ 0.001, post hoc tests *p* ≤ 0.002), except between Sections 2 and 3 in HIIT3m (Figure 3). Furthermore, there was a significant difference between both protocols in the first two sections, where the HIIT3m evoked a higher cardiac output (ANOVA *p* = 0.045, post hoc tests *p* ≤ 0.025).

Cardiac output during the loaded phases was significantly higher (*p* ≤ 0.001) in HIIT3m for all sections. Contrary to the loaded phases, HIIT30s resulted in higher cardiac output in the unloaded phases (*p* ≤ 0.001, Figure 3).

### 3.4. Stroke Volume

Stroke volume remained stable despite the increasing exercise duration, and section averages did not differ significantly between the two protocols. This also applied when data was separated into loaded and unloaded phases (Figure 2 and Figure 3).

### 3.5. Muscle Hemodynamics

HHb/VO_2_ increased from Sections 1–3 (ANOVA *p* = 0.001, post hoc tests *p* ≤ 0.05) in both protocols and stayed stable thereafter (Figure 2). However, there was a significant interaction effect indicating a steeper increase in HIIT30s between Sections 1 and 2 (*p* = 0.027).

Loaded and unloaded phases did not differ significantly between both protocols. But in HIIT30s, there was a significant interaction effect of HHb/VO_2_ between Sections 1 and 2 as well as Sections 2 and 3 in the loaded phases (*p* ≤ 0.05, Figure 3).

In HIIT3m, TSI dropped significantly in response to the first interval (*p* < 0.001), decreased further after the second interval (*p* = 0.012), and remained stable thereafter. This drop was not significant in HIIT30s.

### 3.6. End-Exercise Lactate

End-exercise lactate was significantly (*p* ≤ 0.001) higher in HIIT3m (6.2 ± 2.2 mmol/L) compared to HIIT30s (4.2 ± 2.0 mmol/L).

## 4. Discussion

In order to optimize the effects of training, it is important to know the impact of different training regimens on the desired performance-predicting physiological systems. Our results demonstrate that interval training with long intervals increases training time at high rates of oxygen uptake (i.e., higher aerobic rates) and cardiac output compared to a short interval training at an identical overall workload. This suggests that long-interval training approaches the central cardiovascular system more intensively. Although average responses (i.e., including low intervals) of oxygen uptake and cardiac output did not differ significantly, it agrees with recent findings that the exercise time at high intensities might be crucial for triggering positive effects [25]. Overall metabolism rises disproportionately [24,26], therefore higher aerobic rates represent considerably higher metabolic stimuli, despite the longer unloaded phases during the HIIT3m. This is also supported by much higher lactate values in the HIIT3m.

We have shown in previous studies that isolated 30 s work intervals might lead to lower cardiovascular responses compared to 3 min work intervals [23]. However, repeated 30 s work-interval bouts could nevertheless be effective in a similar way compared to longer interval durations. In the current study, results promote the 3 min intervals to evoke a higher impact on the cardiovascular system. Short-interval training is very often prescribed using short maximal or supramaximal intensities, interspersed by either passive breaks or longer, active relief intervals [1]. This is associated with having a greater effect on VO_2_max development by higher rates of oxygen consumption during training. In the current study, we did not consider this type of interval training as we aimed to focus solely on the effect of interval duration without changing the work rate.

### 4.1. Oxygen Uptake and Central Responses

As mentioned before, long intervals had a higher impact on peak oxygen uptake and peak cardiac output during work intervals. This was associated with the long-lasting demand for oxygen, which is reflected by a plateau or only a minor increase in oxygen uptake at the end of the loaded phases. Work intervals were too short to affect a similar VO_2_ response in HIIT30s and therefore VO_2_ reached lower values without attaining a plateau. On the other hand, VO_2_ dropped lower during resting phases in HIIT3m compared to HIIT30s, resulting in a high amplitude of VO_2_ response to the training session. Contrary, there was only a small variation between loaded and unloaded phases in HIIT30s. Despite this, the average response of VO_2_ to both protocols was similar. Nevertheless, the participants’ aerobic rate did not recover to baseline values in both protocols. Thus, average oxygen uptake increased almost consistently with each interval section.

The high difference in VO_2_ between the loaded and unloaded phases in HIIT3m as well as the small variation in HIIT30s resulted in an equal average response in all sections and protocols. In detail, the HIIT3m elicited shorter training times above GET than the short interval training (1669.9 ± 310.9 s vs. 1769.5 ± 189.0 s, *p* = 0.034) while the average fractional utilization of VO_2_peak was similar (65.23 ± 4.68% VO_2_peak vs. 64.39 ± 6.78% VO_2_peak, *p* = 0.261). However, the long interval training caused a significantly longer time above the target intensity of 80% VO_2_peak (377.3 ± 254.4 s vs. 121.7 ± 370.9 s, *p* = 0.001). Further, 15 subjects exceeded the fractional utilization of VO_2_peak that is corresponding to the occurrence of RCP in the ramp test. In contrast, only 4 subjects reached that level during the short interval training. As oxygen uptake responses to constant load exercise above GET is not only dependent on exercise intensity but also on exercise time due to the oxygen uptake slow component [27], this result is not surprising. However, it indicates higher aerobic flux during the HIIT3m protocol. 8 subjects reached their RCP at intensities of 85% VO_2_peak and higher and therefore did not reach RCP level in fractional utilization of VO_2_peak despite the slow component. These findings indicate that the HIIT3m affects a longer training period in the severe intensity domain. which is typically one major reason for the application of interval regimens in endurance sports.

Directly linked to oxygen uptake, a similar behavior was shown in cardiac output. As a result of incomplete recovery, cardiac output increased in both protocols due to an increase in heart rate, while stroke volume remained constant. Again, the long intervals resulted in higher cardiovascular stress during the loaded phases compared to short intervals. But the section average values of cardiac output showed again a similar impact on the cardiovascular system. However, adjustment of the cardiac output took longer in the long-interval protocol (Figure 3).

### 4.2. Microvascular O_2_ Provision

As previously mentioned, microvascular oxygen provision was estimated by ΔHHb per unit VO_2_. The larger the ratio, the more O_2_ is extracted in relation to VO_2_ uptake. Lower values would indicate a smaller increase of ΔHHb at a given workload by higher local muscle oxygen provision [8,24]. In both protocols, the average ratio of local oxygen supply to systemic demand increased until Section 3, i.e., until the 18th minute of both interval training, and flattened afterward. Local vasodilation could be associated with the improvement of local O_2_ provision after the first three sections. It is assumed that vasoactive substances, like lactate or acetylcholine, might promote tissue perfusion and therefore enhance oxygen uptake on-kinetics, e.g., the response to the work-intervals in this study [24,27,28]. Furthermore, acid substances are known to shift the O_2_ dissociation curve and thus improve muscular O_2_ utilization according to the Bohr Effect [29,30]. However, the Bohr Effect might be of smaller importance in the current study as exercise times in the severe intensity domain were quite short and therefore is not likely to lower local pH that much. Moreover, intermittent contractions during cycling might also enhance blood flow in terms of muscle pumps. Especially during the active rest, where small intensities had to be handled, mean arterial pressure might be maintained constant during the recovery phases compared to a passive rest [31]. However, ΔHHb/VO_2_ increased significantly during the loaded phases in HIIT30s compared to HIIT3m (Figure 3). Although ΔHHb/VO_2_ did not differ following Section 3, HIIT3m presumably promotes vascular effects faster than the HIIT30s.

These results were partially in line with the results of Zafeiridis’ study [11]. Although different intensities and passive rests had been used, no significant differences in local muscle perfusion were detected, while cardiovascular stress was increased by a long interval training. Zafeiridis and colleagues speculated that both long and short intervals trigger microvascular and metabolic adaption due to equal average central responses and similar ΔHHb/VO_2_ [11].

The adaption of interval duration at submaximal workloads is an important parameter to control the cardiorespiratory loading. Reductions in interval length should therefore be accompanied by an increase in workload to achieve a comparable training stimulus for the cardiorespiratory system. The equal work rates might limit the results of the study due to different intensity domains resulting in different metabolic demands. However, interval training affects muscle perfusion independent of interval duration. Thus, short submaximal interval training can be a part of rehabilitation programs to improve muscular perfusion with less cardiac stress. A training study would be necessary to validate the results and estimate long-term adaption effects in the muscle and cardiorespiratory systems.

## 5. Conclusions

In conclusion, the study aimed to detect possible differences in central and peripheral hemodynamics in two interval protocols at the same work rate. Tested short and long interval durations increased average response in oxygen uptake, cardiac output, heart rate and local oxygen provision while stroke volume maintained almost constant. Differences between the protocols were detected regarding loaded and unloaded phases in central hemodynamic parameters and oxygen uptake, where the long-interval training regimen results in stronger cardiovascular and metabolic responses. Therefore, interval duration is a useful tool to adapt interval training to the needs and aims of the training process.

## Figures and Tables

**Table 1:** Subject characteristics.

Parameter	Mean ± SD	N
Age [years]	24.3 ± 3.6	24
Height [cm]	181.4 ± 5.1	24
Weight [kg]	75.9 ± 7.6	24
Skinfold thickness at m. vastus lateralis [mm]	8.0 ± 3.2	24
Peak oxygen uptake (VO_2peak_) [L*min^−1^]	4.11 ± 0.53	24
Relative peak oxygen uptake (VO_2peak_) [mL*min^−1^ *kg^−1^]	54.1 ± 5.3	24
Gas Exchange Threshold (GET) [% VO_2peak_]	52.9 ± 8.4	24
Respiratory Compensation Point (RCP) [% VO_2peak_]	82.6 ± 6.9	24
peak heart rate (HR_peak_) [bpm]	185.0 ± 7.7	24
peak cardiac output (CO_peak_) [L*min^−1^]	25.4 ± 3.4	17
peak stroke volume (SV_peak_) [ml]	144.1 ± 19.4	17
peak power output (PO_peak_) [W]	359.5 ± 44.8	24

## Data Availability

All data, tables and figures presented in this manuscript are original. Further inquiries can be directed to the corresponding author.
